# Outcomes of dietary alpha-lipoic acid on testicular vascularization, steroid hormones, and seminal quality in aged Baladi bucks

**DOI:** 10.1186/s12917-024-04134-4

**Published:** 2024-07-05

**Authors:** Elshymaa A. Abdelnaby, Mohamed Fathi, Noha Y. Salem, Eman S. Ramadan, Shimaa G. Yehia, Ibrahim A. Emam, Ali Salama, Haney Samir, Hossam R. El-Sherbiny

**Affiliations:** 1 Theriogenology Department, Faculty of Veterinary Medicine, Cairo University, Giza, Egypt; 2 Department of Internal Medicine and Infectious Diseases, Faculty of Veterinary Medicine, Cairo University, Giza, 12211 Egypt; 3 Department of Surgery, and Radiology, Faculty of Veterinary Medicine, Cairo University, Giza, Egypt

**Keywords:** Aging, Antioxidants, Alpha-lipoic acid, Doppler, Goats, Semen

## Abstract

**Background:**

Senescence is accompanied by a progressive decrease in male reproductive performance, mainly due to oxidative stress and endothelial dysfunction. Alpha lipoic acid (ALA) is a potent antioxidant, that diffuses freely in aqueous and lipid phases, possessing anti-inflammatory and anti-apoptotic properties. This study aimed to examine the effects of supplemental dietary ALA on testicular hemodynamics (TH), circulating hormones, and semen quality in aged goats. Twelve Baladi bucks were divided into two groups (*n* = 6 each); the first fed a basic ration and served as a control group (CON), while the second received the basic ration supplemented with 600 mg ALA/ kg daily for consecutive eight weeks (ALA).

**Results:**

There were improvements in testicular blood flow in the ALA group evidenced by a lower resistance index (RI) and pulsatility index (PI) concurrent with higher pampiniform-colored areas/pixel (W3-W6). There were increases in testicular volume and decreases in echogenicity (W3-W5; ALA vs. CON). Compared to the CON, ALA-bucks had higher serum concentrations of testosterone, estradiol, and nitric oxide (W3-W5). There were enhancements in semen traits (progressive motility, viability, morphology, and concentration, alanine aminotransferase enzyme) and oxidative biomarkers (catalase, total antioxidant capacity, and malondialdehyde).

**Conclusions:**

ALA dietary supplementation (600 mg/kg diet) improved aged bucks’ reproductive performance by enhancing the testicular volume, testicular hemodynamics, sex steroids, and semen quality.

## Introduction

Males with superior genetic traits are demanded to be in action to the end of their lives, improving animal production outcomes. However, senescence progressively deteriorates organ functions and homeostasis [1]. Aged males suffer a dramatic decrease in reproductive hormones, semen quality, sperm cell concentration, and prolificacy. Many factors integrate into aging pathophysiology including pituitary-testicular axis alterations, hormonal imbalance, vascular impairment, and lipid peroxidation [2–4].

Oxidative stress (OS) is primarily blamed for senescence-associated reproductive dysfunction in male animals [3]. However, the male testicular tissue is equipped with an antioxidant army (enzymatic + non-enzymatic) [5], and advancement in age ruins its synthesis, regeneration, and power, jeopardizing the whole reproductive performance [6–8]. According to several studies, OS has detrimental impacts on animal reproductive performance and functions, including decreased sperm quality and fertility potential [6]. Spermatozoa have low antioxidant protection and large levels of polyunsaturated fatty acids (PUFAs), making them vulnerable to reactive oxygen species (ROS) damage. Alpha-lipoic acid (ALA) is a coenzyme used in mitochondrial metabolism. The reduced form of ALA known as dihydro-lipoic acid (DHLA) is a powerful antioxidant for the mitochondria [9]. ALA and DHLA function as powerful redox couple that can scavenge a range of ROS, including hydroxyl radicals and hypochlorous acid. Numerous reports have demonstrated the effects of dietary ALA supplementation on the antioxidant capabilities of mice and broilers, which also help to improve the quality of meat [10, 11]. Due to its potent antioxidant effects, ALA influences cardiovascular and cognitive health, anti-aging, detoxification, anti-inflammation, anti-cancer, and neuroprotection [11]. ALA decreases oxidative stress, improves neurocognitive performance in rats and dogs, and acts as an anti-inflammatory in mice [12–14].

Since ALA has diversiform actions in the perspective of endothelial functions and OS, as well as energy production, it was hypothesized that dietary ALA could mitigate aging-induced OS and enhance testicular functions. The hypothesis was tested by monitoring the testicular vascularization, echogenic, and biometric changes, reproductive hormones, and semen quality following ALA dietary supplementation in aged bucks.

## Materials and methods

### Ethical committee approval statement

The study was approved by the institutional animal use committee (Vet CU 2009-2022-492) at the Faculty of Veterinary Medicine, Cairo University.

### Animals and housing

Twelve aged Baladi bucks with an average body condition score of 3 with body weight (55 ± 1.5 kg) and age (6.4 ± 0.4 years) were conducted in this experiment. Bucks were routinely scanned by B-mode and colored Doppler ultrasound to exclude any bucks suffering any andrological and cardiovascular diseases. They were housed in a paddock enjoying daylight with a 25 m^2^ shaded sector. Animals were provided a daily balanced diet depending on NRC requirements as the normal diet consisted of 500 g/animal every day in the form of pelleted concentrates that contain crude protein (15–19%) with 1.34 kg /animal every day in the form of roughage with fresh water available all day.

### Experimental design, location, and time points

The present study was performed at the small ruminant research farm belonging to the Department of Theriogenology, Faculty of Veterinary Medicine, Cairo University. Bucks were divided into two main groups, the group I (*n* = 6, control bucks with age and weight of 6.4 ± 0.4 years and 55 ± 1.6 kg respectively) received the basic diet only; group II (*n* = 6, ALA bucks with age and weight of 6.2 ± 0.4 years and 50 ± 1.5 kg, respectively) received the basic diet supplemented with ALA (600 mg/kg diet; Thiotacid compound 600, Eva Pharma co., Egypt) for eight wks (W1-W8; goat spermatogenesis) [15, 16]. Bucks were examined (ultrasound, semen, and biochemical analyses) on the first day of supplementation (W 0) and once/week (W1-W8).

### Testicular ultrasonography

#### Biometry and Echogenicity

B-mode linear probe (7.5 MHz; EXAGO, Echo Control Medical, France) was used to measure testicular volume and echogenicity (TE). Testicular volume was estimated following the equation for ellipsoid = 4/3π abc, where (a) for testicular length/2, (b) for width/2, and (c) for height/2 [17], while both TE [pixel heterogeneity (PH) and testicular echotexture (TE)] were assessed using the Adobe Photoshop CC programme as previously published [18, 19].

#### Testicular vascular perfusion (TVP)

TVP was measured using color-Doppler ultrasound (7.5 MHz; EXAGO, Echo Control Medical, France). Bucks were controlled without analgesics and the scrotal hair (including the spermatic cord area) was clipped and shaven. The STA was visualized (B-mode; at the proximal end of the testis) meshed with the pampiniform plexus followed by activating the color mode showing the blue area (testicular direction) and the red area (probe direction) [20]. Pulsed-wave Doppler mode was then activated for recording the arterial cardiac cycle as previously described [21]. The device settings were as follows: window gate (0.5 mm), color maps (2), brightness (56%), pulse repetition frequency (3000), and insonation angle (< 60°). The examined Doppler parameters were resistance (RI) and pulsatility indices (PI).

### Hormonal and nitric oxide assay

Just before the ultrasound examination (9:00 AM), blood samples were collected (jugular vein) into vacutainer tubes (4 ml), centrifuged (15 min at 3000 rpm), harvested, and preserved at − 18 °C for hormonal analysis in the sera. Follicle-stimulating hormone (FSH; ng/mL), luteinizing hormone (LH; ng/mL), testosterone (T; ng/mL), and estradiol (E2; pg/mL) were measured with an intra- and inter-assay variation coefficient of 10 and 12%, respectively using commercial ELISA kits (Sun Long Biotech Co., LTD CHINA). NO metabolites (nitrate and nitrites) were measured photometrically (spectrophotometer; 540 nm) by commercial kits (Bio-diagnostics co., Tahrir st., Giza, Egypt) for representing the serum NO concentrations with an inter-assay variation coefficient of 6.9% and a sensitivity of 0.225 µmol/l [22, 23].

### Semen picture assessment

Semen was extracted from clean, sterile, prewarmed (37 ^o^C) falcon tubes using an electro-ejaculator and kept at that temperature for further examination. Each sample was divided into two portions, one for the evaluation of sperm quality and the other for the biochemical study of seminal plasma (SP). SP was extracted using a sterile pipette, centrifuged at 2000 g for 15 min at 4 oC, and then kept at -20 oC for subsequent analysis [24].

#### Sperm traits

Sperm progressive movement (SPM, %) was examined by placing a 10 µl diluted (1:30 v/v, sodium citrate dihydrate 2.9%) semen drop on a prewarmed (37 ^o^C) clean, dry, and sterile glass slide, cover-slipped, and visualized utilizing phase-contrast microscopy (heated stage: 37 ^o^C; magnification: 400 x). At different representable microscopic fields, the percent of forward and rectilinear motile spermatozoa was recorded. Two slides/buck were examined and averaged for data verification.

Supra-vital eosin-nigrosin staining method was adopted for the evaluation of sperm viability (SV) and normal morphology (NS). In 100 ml double distilled water, 1.67 g eosin, 10 g nigrosin, and 2.9% sodium citrate dihydrate were dissolved (boiling water bath) and filtered (three times) [24]. A diluted semen sample was mixed with a triple amount of the stain, smeared, and air-dried. For SV %, a sum of 300 sperm/slide in different microscopic areas was examined for the stained (partially or completely; dead) and unstained (viable) spermatozoa and recorded in percent. For NS, the SV slide was examined for different sperm abnormalities including head (size and shape) and tail (protoplasmic droplets, coiled, tapered, bent) defects. The results are expressed as the percentage of normal spermatozoa. Sperm cell concentration (SCC, 10^9^ sperm/ml) was examined using an improved Neubauer hemocytometer following a previous report [25].

#### SP oxidative biomarkers and ALT activity

To collect the seminal plasma, the semen samples were spun at a force of 2000 g at a temperature of 4 °C for 15 min. Afterward, the resultant supernatant was gathered was kept at a temperature of -20 °C until it was time for further analysis. Enzymatic activity of catalase (CAT) (U/L), Alanine aminotransferase (ALT) (U/ml), and levels of malondialdehyde (MDA) (mM/ml) and total antioxidant capacity (TAC) (mM/L) were analyzed utilizing a spectrophotometer (commercial colorimetric kits) with the guidance of the manufacturer’s instructions (Bio-diagnostics co., Tahrir st., Giza, Egypt), specifically at a wavelength of 520, 505, 534, and 510 nm, respectively.

### Statistical analysis

Firstly, Shapiro-Wilk and Levene tests were used to determine data normal distribution and homogeneity, respectively. Since the ultrasound parameters didn’t differ between the right and left testis, the data of both testes were pooled and the means for each time point were shown. The differences between control and ALA males (treatment effect) in testicular vascularization values (PI, RI, and CA), circulating hormones (T, E2, FSH, and LH) and NOMs, semen traits (SPM, SV, NS, and SCC), and SP biomarkers (MDA, TAC, CAT, and ALT) during the examined time points (W0-W7; time effect) with the interaction between treatment and time were analyzed using repeated measure two- way ANOVA using the statistical package SPSS 20.

## Results

### B-mode ultrasonographic measurements

**Fig. 1:**
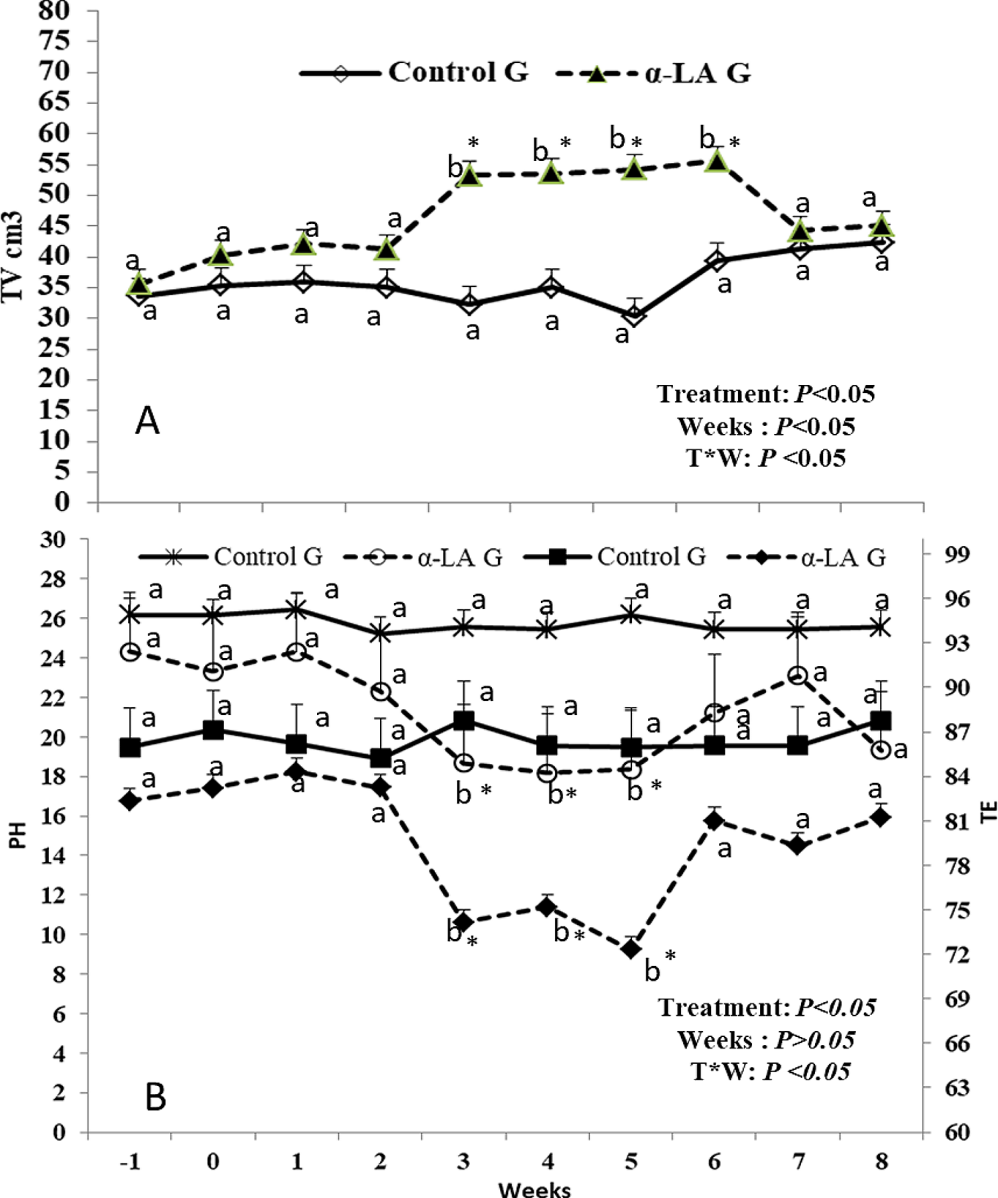
Testicular volume (**A**) and testicular echotexture (**B**) in the form of pixel heterogeneity (PH, SdNPVs) and testicular echogenicity (TE, NPVs). ^a, b^ values are significantly different at *P* < 0.05 compared with the control and α lipoic acid (ALA) males, while the * value is significantly different at *P* < 0.05 between the two groups at the same time point. T = treatment, and W = weeks

ALA dietary supplementation affected both testicular volume (cm^3^) as well as testicular echotexture, as testicular volume increased significantly (*P* < 0.05) from week 3 (53.24 ± 1.22) to week 6 (55.66 ± 1.85). In addition, the time and interaction between time with treatment had shown a significant (*P* < 0.05) difference in testicular volume in the ALA group compared to the control group (Fig. 1A). echogenic changes of the testicular parenchyma (TE and PH) were affected by treatment and time*treatment interaction (*P* < 0.05) by declination from week 3 (74.14 ± 1.02 for TE; 18.66 ± 0.33 for PH) till week 5 (72.31 ± 1.22 for TE; 18.01 ± 0.21 for PH) as shown in (Fig. 1B).

### Testicular blood flow alterations

Values of Doppler indices (resistance and pulstatility [RI, PI]) and CA of the STA were affected by supplementation, weeks, and supplementation*weeks (*P* < 0.05; Table 1). Instantly, RI values decreased significantly (*P* < 0.05) in the ALA-supplemented males from W3 (0.71 ± 0.02) to W6 (0.61 ± 0.02) compared to the controls (0.91 ± 0.01 to 0.89 ± 0.01, respectively). In addition, PI means of the STA declined significantly (*P* < 0.05) from W3 (0.45 ± 0.02) until W6 (0.42 ± 0.02) compared to the control group (0.71 ± 0.02 to 0.71 ± 0.02). The colored areas (testicular direction) elevated significantly (*P* < 0.05) in the ALA-supplemented bucks from W3 (987.25 ± 23.65) until W6 (1165.02 ± 17.36) compared to the controls (595.21 ± 33.62 to 788.32 ± 11.87).

**Table 1:** Values of the supra testicular artery Doppler indices (PI, RI, and CA/testes) in the α-LA versus control (*n* = 6 each) aged bucks throughout the study timepoints (W0-W8)

W	PI		RI		CA/testes (pixel)	
	ALA	CON	ALA	CON	ALA	CON
W0	0.84 ± 0.01	0.88 ± 0.01	0.71 ± 0.01	0.73 ± 0.02	586.25 ± 15.64	666.25 ± 22.15
W1	0.86 ± 0.01	0.92 ± 0.01	0.65 ± 0.01	0.69 ± 0.01	612.25 ± 13.55	654.23 ± 13.25
W2	0.84 ± 0.02	0.88 ± 0.01	0.65 ± 0.01	0.69 ± 0.02	655.21 ± 24.54	687.22 ± 18.65
W3	0.63 ± 0.02*	0.89 ± 0.01	0.45 ± 0.02*	0.73 ± 0.02	987.25 ± 23.65*	687.25 ± 13.25
W4	0.64 ± 0.02*	0.90 ± 0.01	0.43 ± 0.02*	0.72 ± 0.01	1025.25 ± 11.65*	766.5 ± 35.12
W5	0.61 ± 0.02*	0.86 ± 0.01	0.41 ± 0.02*	0.72 ± 0.02	1132.62 ± 24.31*	866.61 ± 11.32
W6	0.66 ± 0.02*	0.87 ± 0.01	0.42 ± 0.02*	0.73 ± 0.02	1165.02 ± 17.36*	883.25 ± 12.61
W7	0.84 ± 0.01	0.88 ± 0.02	0.68 ± 0.01	0.71 ± 0.02	866.25 ± 22.01	889.32 ± 21.32
W8	0.86 ± 0.01	0.89 ± 0.01	0.69 ± 0.01	0.73 ± 0.02	766.25 ± 14.65	788.32 ± 11.87

RI = resistive index, PI = pulsatility index, W = weeks, CA = colored areas (pixels), W = weeks, α-LA = alpha lipoic acid, and CON = control group. *Values are different at least at *P* < 0.05 between the two groups. Data are expressed as mean ± standard error of the mean. There were significant effects of supplementation, weeks, and supplementation* weeks (*P* < 0.05 each) in the values of PI, RI, and CA/testes

### Hormonal levels and semen picture alterations

**Fig. 2:**
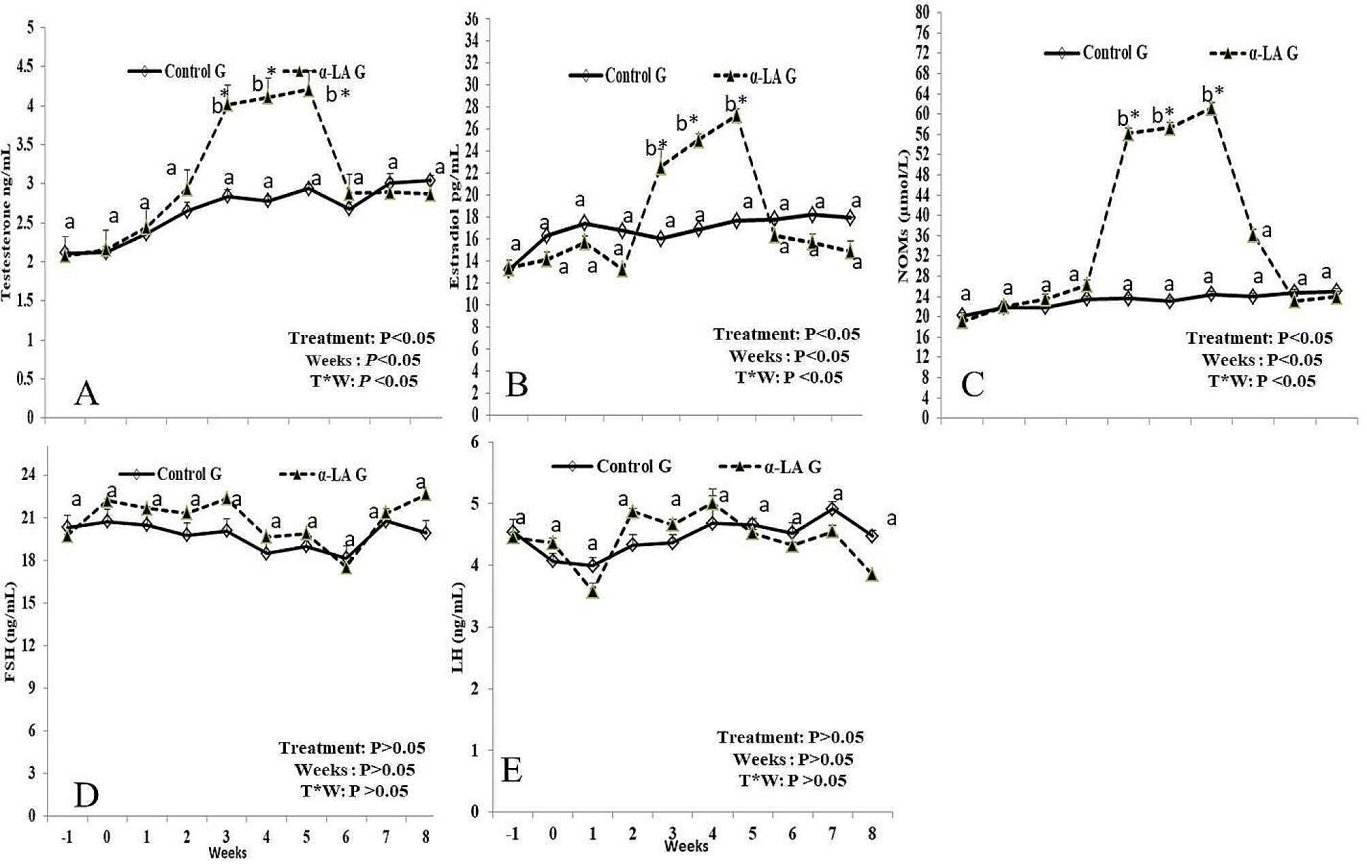
Serum levels of testosterone (T; ng/mL; **A**), estradiol (E2; pg/mL; **B**), nitric oxide (NOMs, µmol/L; **C**), follicle-stimulating hormone (FSH; ng/mL; **D**), and luteinizing hormones (LH; ng/mL; **E**) in male bucks administrated α-lipoic acid (ALA) compared to control group. Values are presented as means ± SEM. ^a, b^ values are significantly different at *P* < 0.05 compared with the control and ALA males, while the * value is significantly different at *P* < 0.05 between the two groups at the same time point. T = treatment, and W = weeks

Serum concentrations of testosterone (T), estradiol (E2), and nitric oxide (NO) were affected by supplementation, weeks, and supplementation*weeks (*P* < 0.05; Fig. 2A, B, and C). In detail, serum levels of T and E2 elevated (*P* < 0.05) during W3 (4.02 ± 0.04 ng/mL for T and 22.54 ± 1.44 pg/mL for E2) till W5 (4.21 ± 0.04 ng/mL for T and 27.23 ± 1.34 pg/mL for E2). In addition, NO levels increased (*P* < 0.05) from W3 (56.23 ± 1.33 µmol/L) to W5 (61.21 ± 1.32 µmol/L). The ALA supplementation did not affect FSH and LH (Fig. 2D and E).

The semen parameters (SPM, SV, NS, and SCC) in ALA-supplemented and control bucks is presented in Table 2. There were effects (*P* < 0.05) of supplementation, week, and supplementation*weeks interaction in SPM, SV, and SCC; while NS was only affected (*P* < 0.05) by supplementation and supplementation*week interaction. Precisely, ALA supplementation increased significantly (*P* < 0.05) the percentage of SPM, SV, and NS (W5-W8), while SSC elevated (*P* < 0.05; W6-W8).

**Table 2:** Values of the semen traits (progressive motility, viability, normal morphology, and sperm concentration) in the α-LA-supplemented versus control (*n* = 6 each) aged bucks during the study timeline (W0-W8)

W	Progressive motility (%)		Viability (SV;%)		Normal morphology (SPM;%)		Sperm cell concentration 10^9^/Ml (SCC)	
	ALA	CON	ALA	CON	ALA	CON	ALA	CON
W0	71.21 ± 1.14	69.00 ± 1.44	81.40 ± 1.01	82.20 ± 1.44	83.60 ± 2.66	80.20 ± 1.61	2.28 ± 0.37	2.33 ± 0.51
W1	72.13 ± 1.34	69.00 ± 1.44	78.70 ± 1.84	80.40 ± 1.44	81.30 ± 0.81	82.80 ± 0.74	2.25 ± 0.07	2.22 ± 0.11
W2	71.20 ± 1.11	70.00 ± 1.75	80.60 ± 1.22	78.40 ± 0.98	83.00 ± 0.58	83.40 ± 0.25	2.20 ± 0.27	2.27 ± 0.35
W3	70.45 ± 1.75	69.00 ± 1.44	79.40 ± 1.24	80.80 ± 1.11	82.33 ± 0.65	82.80 ± 0.74	2.17 ± 0.37	2.22 ± 0.11
W4	70.00 ± 1.04	69.00 ± 1.44	78.20 ± 1.44	80.17 ± 1.01	82.40 ± 0.87	82.80 ± 0.74	2.23 ± 0.13	2.19 ± 0.11
W5	82.00 ± 2.14*	74.00 ± 1.88	90.80 ± 1.84*	81.60 ± 1.01	91.87 ± 0.55*	84.80 ± 0.12	2.28 ± 0.32	2.33 ± 0.51
W6	83.20 ± 1.12*	73.00 ± 1.88	92.80 ± 1.20*	79.50 ± 1.11	93.20 ± 0.41*	82.70 ± 0.01	2.55 ± 0.02*	2.17 ± 0.02
W7	81.00 ± 2.11*	74.00 ± 1.88	90.80 ± 1.84*	81.60 ± 1.01	91.80 ± 0.95*	84.80 ± 0.12	2.66 ± 0.01*	2.10 ± 0.01
W8	84.00 ± 1.74*	74.00 ± 1.04	89.80 ± 1.77*	80.10 ± 1.44	89.20 ± 0.44*	84.55 ± 0.15	2.61 ± 0.03*	2.15 ± 0.02

W = weeks, ALA = alpha lipoic acid, and CON = control group. There were effects (*P* < 0.05) of supplementation, week, and supplementation*weeks interaction in SPM, SV, and SCC; while NS was only affected (*P* < 0.05) by supplementation and supplementation*week interaction. *Values are different at least at *P* < 0.05 between the two groups

### SP oxidative biomarkers and ALT activity

Data of SP levels of CAT, TAC, ALT, and MDA in the ALA-supplemented and control bucks through the study time frame (W0-W8) are presented in Table 3. The supplementation*week effects showed significant (*P* < 0.05) differences in MDA, TAC, CAT, and ALT, while the time effect showed a significant (*P* < 0.05) difference in CAT and ALT activities. In detail, TAC activity increased significantly starting from week 4 and this pattern sustained till week 8, same pattern was recorded in catalase activity at week 5 and continue till end of expermint (week 8). MDA activity starts to decrease significantly beginning from week 6 and this pattern sustained till week 8, while ALT level start to decline starting from week 5 and continue till week 8.

**Table 3:** Mean ± standard error of the mean (SEM) of seminal plasma levels of MDA, TAC, CAT, and ALT enzymes in α-lipoic acid supplemented bucks (ALA; *n* = 6) group at the studied time points versus the control (CON; *n* = 6) group at the different time points (W0-W8)

W	MDA (nM/mL)		TAC (mM/L)		CAT (U/L)		ALT (U/mL)	
	ALA	CON	ALA	Cont	ALA	CON	ALA	CON
W0	2.86 ± 0.01	2.71 ± 0.11	0.60 ± 0.01	0.95 ± 0.01	288.19 ± 24.33	271.71 ± 22.31	46.17 ± 0.98	43.22 ± 0.22
W1	2.74 ± 0.01	2.71 ± 0.12	0.64 ± 0.01	0.61 ± 0.01	265.18 ± 27.01	281.24 ± 21.27	46.58 ± 1.02	45.32 ± 1.84
W2	2.78 ± 0.01	2.74 ± 0.01	0.67 ± 0.03	0.68 ± 0.01	237.32 ± 26.32	258.21 ± 25.25	46.40 ± 1.44	43.99 ± 1.77
W3	2.77 ± 0.01	2.75 ± 0.01	0.62 ± 0.02	0.62 ± 0.01	251.91 ± 25.32	299.21 ± 31.25	46.84 ± 1.32	44.01 ± 2.74
W4	2.63 ± 0.02	2.77 ± 0.04	1.05 ± 0.01*	0.78 ± 0.02	288.26 ± 24.66	312.02 ± 44.11	46.55 ± 2.01	43.25 ± 1.77
W5	2.68 ± 0.04	2.74 ± 0.03	1.33 ± 0.01*	0.88 ± 0.06	380.45 ± 22.32*	288.54 ± 21.32	38.33 ± 0.25*	44.04 ± 0.99
W6	2.20 ± 0.03*	2.61 ± 0.01	1.34 ± 0.05*	0.91 ± 0.04	437.91 ± 12.65*	311.25 ± 31.02	40.21 ± 1.14*	45.40 ± 2.95
W7	2.37 ± 0.02*	2.61 ± 0.01	1.29 ± 0.02*	0.92 ± 0.08	525.57 ± 17.32*	301.21 ± 37.25	35.26 ± 0.51*	43.75 ± 2.44
W8	2.29 ± 0.05*	2.72 ± 0.02	1.42 ± 0.05*	0.98 ± 0.07	492.24 ± 18.35*	340.12 ± 25.23	31.45 ± 0.24*	42.5 ± 1.47

MDA = malondialdehyde, TAC = total antioxidant capacity, CAT = catalase activity, ALT = alanine aminotransferase, and W = weeks. The supplementation*week effects showed significant (*P* < 0.05) differences in MDA, TAC, CAT, and ALT, while the time effect showed a significant (*P* < 0.05) difference in CAT and ALT activities.*Values are different at least at *P* < 0.05 between the two groups

## Discussion

Males possessing superior genetic merits have pivotal impacts on animal breeding success even at senescence. Therefore, this work aimed to alleviate the adverse effects of senescence on testicular vascularization and subsequent steroidogenic and spermatogenic functions by dietary supplementation of ALA. These outcomes may help the animal breeding industry to improve the use of superior males till the last day of their lives. Present work reported a decrease in RI and PI values of the STA concurrent with an increase in pampiniform plexus CA in the ALA-supplemented (W3-W6) compared to the unsupplemented group. Lower RI and PI, and higher CA values indicate an improvement in the blood flow within the STA by decreasing the arterial resistance and ultimately higher blood perfusion [26–28]. Despite the actual mechanisms by which ALA enhanced the TBF were not examined here, different explanations could ease the vision. To begin with, ALA protects vascular and endothelial functions by influencing the NO-mediated vasodilation pathway and being an antithrombotic agent [29]. This evidence supports the higher levels of serum NOMs levels observed in the present study. Aging is mainly accompanied by vascular and endothelial irregularities due to oxidant/antioxidant imbalance. In addition, ALA enhances vascular homeostasis by altering endothelial responsiveness [30] and modulating angiogenic factors [31]. Furthermore, being a potent ROS scavenging molecule, ALA increases NO bioavailability through coupling with superoxide anions (main endothelial deteriorative radical) [32].

ALA-supplemented bucks showed higher TV measurements and lower echogenicity through W3 to W5 compared to the controls, indicating a testicular flourishment. Surprisingly, the changes in TV and TE were parallel to the improvement in testicular hemodynamics. Higher TBF is positively correlated with TV and negatively with TE [33]. Indeed, a study reported an increase in the TV of varicocelised rats by oral ALA supplementation through reversing the negative impacts of varicocele on testicular tissue [34]. In addition, supplemental dietary ALA improved testicular histomorphometric parameters evidenced by higher epithelial height and wider seminiferous tubules in senile breeder roosters [35].

ALA-supplemented bucks possessed higher serum concentrations of T and E2 (W3-W5) compared to controls. These results are corroborated by a recent study on aged breeder roosters [35] that reported a *caspase 3* downregulation concurrent with upregulation of *Nrf2* mRNA in the testicular tissue which affirms the anti-apoptotic properties of ALA [35]. ALA-antioxidant capacity is beyond special, as it not only compulsively scavenges free radicals but also can regenerate endogenous antioxidant defense (glutathione, vitamins A, C, and E) being a super-protector for the steroids’ producing cells (Leydig and Sertoli) [36]. Though the FSH and LH serum levels were not significantly altered by ALA supplementation, the T and E2 improvements should be interpreted cautiously as the local role of ALA in the testes couldn’t be negated [37, 38].

Assessment of semen traits unveils the actual andrological properties of males [39]. ALA-bucks semen quality witnessed improvements in sperm progressive motility, viability, morphology, and concentration as well as SP oxidative biomarkers (CAT, TAC, and MDA). These results are supported by many studies on men [40, 41], rodents [37], and Roosters [35]. Goat spermatozoa possess higher levels of PUFAs making them highly susceptible to membrane damage via lipid peroxides [42, 43]. ALA can make a protective shield covering the spermatozoa making them less injured by ROS [4]. ALA is a mitochondrial coenzyme integrating into ATP production that affects sperm motility and hyperactivity [44].

In the current investigation, ALA supplementation caused a significant reduction in MDA and elevation in TAC, similar findings were reported in infertile men received ALA supplementation [41]. ALA showed to reduce production of ROS and inhibit lipid peroxidation which in turn, provides a protective effect for goat sperms [45].

In the present study, catalase activity start to elevate from week 5, in one study investigate the effect of ALA supplementation on antioxidant enzymes on semen in cashmere goat, there was elevation of CAT level [46]. The higher enzymatic activity (reported herein) of CAT and TAC and lower MDA and ALT ascertain the protective role of ALA against oxidative stress and membrane lipid peroxidation. Many studies have reported improvements in the seminal antioxidant defense by ALA supplementation in different stress conditions and species either in vivo or in vitro [35, 37, 47]. Research on the oxidation of phospholipids within mammalian sperm indicates that this oxidative process results in damage to the membrane, ultimately causing a decrease in the ability of sperm to move effectively [48]. In one human study, the concentration of total antioxidant capacity in the seminal fluid of men with asthenozoospermia was found to be considerably less compared to that of healthy individuals. Furthermore, a direct association was observed between diminished TAC concentrations and decreased movement of sperm [49]. Measuring the activity of alanine transaminase (ALT) is deemed a valuable indicator of the integrity of the sperm plasma membrane. This is due to the fact that sperm cells with compromised membranes tend to discharge ALT into the seminal fluid [50]. The vascular supply is expressed by Lower RI and PI, and higher blood flow expressed by elevation of colored area with increase the voulme of the main artery [28, 51–53], as ALA is recomeended to improve the sexual activity, fertility potential, and testicular blood flow.

## Conclusion

Alpha lipoic acid dietary supplementation ameliorated the adverse impacts of senescence of testicular blood flow, sex hormones (testosterone and estradiol), semen quality, and oxidative biomarkers. Newly proposed studies are demanded to investigate the effect of dietary ALA on advanced sperm evaluation techniques, sexual activity, and fertility potential.

## Data Availability

The raw data support outcomes of the present study is available by the corresponding author.
